# Pathological Characteristics of the Lung and Brain in Cotton Rats and BALB/c Mice Infected with Respiratory Syncytial Virus

**DOI:** 10.3390/v18030382

**Published:** 2026-03-18

**Authors:** Ziou Wang, Bowei Jiang, Zhen Huang, Miao Liu, Zheli Li, Weihu Long, Hong Shen, Shengtao Fan, Yousong Ye, Zhangqiong Huang

**Affiliations:** Institute of Medical Biology, Chinese Academy of Medical Sciences & Peking Union Medical College, No. 935, Jiaoling Road, Kunming 650118, China; wzo@pumc.edu.cn (Z.W.); s2025018028@student.pumc.edu.cn (B.J.); s2024018030@student.pumc.edu.cn (Z.H.); s2024018028@student.pumc.edu.cn (M.L.); lzl@imbcams.com.cn (Z.L.); longweihu@imbcams.com.cn (W.L.); shenhc@student.pumc.edu.cn (H.S.); fst@imbcams.com.cn (S.F.)

**Keywords:** respiratory syncytial virus, cotton rats, BALB/c mice, lung, brain, central nervous system, microglia

## Abstract

To compare the respiratory lesions and nervous system damage in cotton rats and BALB/c mice following respiratory syncytial virus (RSV) infection, and to evaluate their suitability as models for RSV-related respiratory and nervous system diseases, cotton rats and BALB/c mice were infected with RSV via intranasal instillation, monitored daily for weight and temperature. At 3, 5, and 7 days post-infection (dpi), viral loads in the nasal turbinates, lungs, and brain tissues were quantified. Pathological changes and neuroinflammatory responses in the lungs and brain were assessed using hematoxylin and eosin (H&E) staining, Nissl staining, immunofluorescence, and Western blotting analysis, while the mRNA expression levels of inflammatory factors were specifically analyzed at 5 dpi. The results showed that viral loads in the nasal turbinates and lungs of cotton rats were significantly higher than those in BALB/c mice, accompanied by more extensive pulmonary inflammatory factor gene upregulation at 5 dpi and more pronounced lung histopathological alterations. In contrast, RSV RNA and antigens were detected in the brain tissues of BALB/c mice, at levels markedly lower than those in respiratory tissues, along with viral antigens primarily localized to the choroid plexus epithelium. No significant pathological or neuroinflammatory changes were observed in the brains of cotton rats at any examined time point. In conclusion, cotton rats provide advantages for modeling RSV-associated respiratory tract infection and pulmonary pathology, whereas under the experimental conditions of this study, BALB/c mice may be more appropriate for investigating RSV-associated CNS inflammatory responses, although the clinical relevance of these findings remains to be further validated.

## 1. Introduction

Respiratory syncytial virus (RSV) is a negative-sense single-stranded RNA virus that belongs to the genus *Orthopneumovirus* within the family *Pneumoviridae* [1]. Human respiratory syncytial virus (hRSV) is a significant pathogen responsible for acute lower respiratory tract infections (ALRTI) in neonates, children, and older adults. Approximately 33.8 million ALRTI cases occur in children under five years of age annually, resulting in approximately 3.4 million hospitalizations [2,3]. One prospective study encompassing 110,058 patients with acute respiratory infections in China revealed an RSV detection rate of 16.8%, ranking second among all viruses [4]. Common clinical manifestations of hRSV infection include cough, wheezing, fever, nasal congestion, sore throat, and vomiting, with severe cases progressing to bronchitis, pneumonia, and respiratory failure [5]. In recent years, substantial progress has been made in RSV prevention. Prefusion F protein–based vaccines, including Arexvy for older adults [6] and Abrysvo for both older adults and maternal immunization during late pregnancy [7], as well as the long-acting monoclonal antibody nirsevimab for infants [8], have been approved in multiple regions. Despite these prophylactic advancements, important questions remain regarding RSV pathogenesis, particularly extrapulmonary complications. Consequently, appropriate animal models remain essential for characterizing disease progression and host responses and for providing an experimental basis for future research.

However, hRSV is highly specific to humans, and no animal model can fully replicate the upper and lower respiratory tract diseases that follow infection [9,10]. Mouse and cotton rat models of infection are the most commonly used animal models for the development of RSV vaccines and drugs. The susceptibility of cotton rats to RSV in the respiratory system is approximately 100 times that of BALB/c mice [11,12]. Additionally, neutralizing antibody levels in cotton rats following natural infection or immunization are typically comparable to those reported in human infants and remain susceptible to RSV into adulthood, making them a useful model for long-term studies [13,14]. However, relatively few experimental tools are available for cotton rat studies.

The BALB/c mouse is a semi-permissive host for RSV replication, and higher intranasal inoculation doses are often used to provoke signs of lower respiratory tract infection [15]. The BALB/c mouse model remains highly valuable for studying the immunological mechanisms of RSV infection [16]. For example, this mouse model has been widely used to investigate immune responses following RSV infection, particularly the balance of Th1/Th2-type immune responses and the cytotoxic T lymphocyte (CTL) response [17,18,19]. Cotton rats and BALB/c mice exhibit physiological differences, particularly in their immune systems, leading to distinct response patterns following RSV infection. Therefore, animal models should be chosen in accordance with the research purpose.

Recent studies have shown that some severe pediatric respiratory infections can damage the nervous system, leading to cognitive impairments in addition to inflammatory responses in severe cases [20,21]. Saravanos et al. systematically reviewed studies of patients under 15 years of age with severe acute neurological complications associated with RSV. Across the 87 studies included, seizures, encephalitis, and encephalopathy were the most common neurological complications [22]. RSV-induced encephalopathy may result from the direct invasion of the central nervous system (CNS) by the virus or indirect mechanisms mediated by circulating molecules such as cytokines and metabolites [23]. Previous studies using mouse models have confirmed that RSV can infect olfactory sensory neurons and directly invade the CNS by entering the olfactory bulb [24,25]. One study has demonstrated that RSV can infect neural cells and induce neural damage through the lung–brain axis [26]. However, the neuropathological features of RSV infection remain incompletely explored, and research on related neurological complications remains relatively scarce. Further investigation into the relationship between RSV infection and neurological complications is important for improving our understanding of the clinical spectrum of RSV infection, optimizing therapeutic strategies, and developing effective preventive measures. An appropriate animal model is essential for studying RSV-associated neurological complications and their underlying mechanisms. Few studies have focused on RSV-associated neurological diseases, and existing studies have employed BALB/c mice as the main model. Most RSV studies using cotton rat models have focused on respiratory infection, pulmonary pathology, or the evaluation of vaccines and antiviral agents [27,28,29]. To our knowledge, studies investigating RSV-associated neurological manifestations in cotton rat models remain extremely limited.

This study was conducted to systematically compare the susceptibility of cotton rats and BALB/c mice to RSV infection in the respiratory system and CNS, and to examine the associated pathological changes in the lungs and brains of these two models. This comparative analysis was intended to evaluate the respective strengths and limitations of each model for studying RSV-related respiratory disease and CNS-associated responses.

## 2. Materials and Methods

### 2.1. Antibodies

The anti-β actin antibody was obtained from Proteintech (Wuhan, China, catalog # 20536-1-AP), the HRP-conjugated anti-rabbit IgG antibodies from Cell Signaling Technology (Boston, MA, USA, catalog # 7074S) and Boster (Pleasanton, CA, USA, catalog # BA1055), the anti-RSV antibody from Abcam (London, UK, catalog # ab43812), the anti-GFAP antibody from Abcam (London, UK, catalog # ab68428), the anti-Iba1 antibodies from Abcam (London, UK, catalog # AB178846) and Boster (Pleasanton, CA, USA, catalog # M01394), and Alexa Fluor 488-conjugated goat anti-rabbit IgG from Servicebio (Wuhan, China, catalog # GB25303).

### 2.2. Animals

Specific pathogen-free (SPF) female BALB/c mice aged 4–6 weeks (body weight approximately 15–20 g) were purchased from the Laboratory Animal Department of the Institute of Medical Biology, Chinese Academy of Medical Sciences (Kunming, China) [SCXK (Dian) 2022-0002]. SPF female cotton rats aged 4–6 weeks (body weight approximately 125–135 g) were obtained from SPF (Beijing) Biotechnology Co., Ltd. [SCXK (Jing) 2024-0001] (Beijing, China). The mice and cotton rats were housed in a barrier environment (20–24 °C; 30–70% humidity; 12 h light/dark cycle) [SYXK (Dian) 2022-0006] at the Laboratory Animal Department of the Institute of Medical Biology, Chinese Academy of Medical Sciences, and were provided free access to water and food. The experimental protocol was reviewed and approved by the Institutional Animal Care and Use Committee of the Institute of Medical Biology, Chinese Academy of Medical Sciences (DWSP202409006). The 3R principles of animal use were followed throughout the experimental process to ensure humane treatment of the animals.

### 2.3. Animal Anesthesia and Euthanasia

In most experiments, isoflurane (RWD Life Science, Shenzhen, China) gas was used for anesthesia as follows: the animals were placed in an induction chamber containing 4–5% isoflurane, delivered via oxygen. Behavioral changes were monitored, and successful anesthesia induction was confirmed when the animals exhibited closed eyes and loss of voluntary movement. In some experiments, 1.25% Avertin (Nanjing Jiancheng Bioengineering Institute, Nanjing, China) was injected intraperitoneally at 0.2 mL/10 g body weight to achieve deep anesthesia.

Carbon dioxide (CO_2_) was utilized for euthanasia as follows: the animals were placed in a sealed euthanasia chamber, after which CO_2_ was introduced at a flow rate of 10–30% of the chamber volume per minute. Death was confirmed as cessation of respiration and heartbeat. Throughout the experimental process, all procedures were conducted strictly in accordance with animal ethics guidelines to ensure the mice remained free from pain and distress.

### 2.4. Cells and Virus

Hep-2 cells were purchased from Boster (Wuhan, China) and cultured in Dulbecco’s Modified Eagle Medium-Nutrient Mixture F-12 (DMEM–F12) (Gibco, Grand Island, NY, USA) supplemented with 10% fetal bovine serum (Gibco, Grand Island, NY, USA) at 37 °C in a 5% CO_2_ atmosphere. The RSV strain 1540 was kindly provided by Dr. Li Qianqian’s laboratory at the Institute of Medical Biology, Chinese Academy of Medical Sciences, and maintained in our laboratory.

### 2.5. Virus Amplification and Titration

Hep-2 cells at 80–90% confluency were infected with RSV at a multiplicity of infection (MOI) of 0.1 and incubated at 37 °C for 2 h to allow virus adsorption. Subsequently, 14 mL of virus maintenance medium (2% fetal calf serum) was added, and the cells were cultured in an incubator. The cytopathic effect (CPE) was monitored daily. The virus was harvested when 80–90% of the cells in the culture flask exhibited CPE. The culture flask was then subjected to two cycles of freeze–thawing at −80 °C, followed by centrifugation at 2000 rpm for 15 min to remove cell debris. The clarified viral supernatant was aliquoted into 1.5 mL Eppendorf tubes, with 1 mL per tube, and stored at −80 °C for subsequent experiments.

The Hep-2 cell suspension was diluted to 1 × 10^5^ cells/mL, and 100 μL of the diluted cell suspension was added to each well of a 96-well plate. The plate was then incubated in a CO_2_ incubator for 24 h. The virus suspension to be tested was serially diluted 10-fold in virus dilution medium to obtain dilutions ranging from 10^−1^ to 10^−10^. The 96-well plate was removed, the medium was aspirated, and the wells were washed three times with PBS. One hundred microliters of the diluted virus suspension were added to each well, with the last two rows of the plate serving as normal controls and receiving 100 μL of pure medium. The plate was incubated in a CO_2_ incubator, and the wells were regularly examined for signs of infection. The experiment was terminated when significant cytopathic effects were observed, and the infection status of each well was recorded. The TCID_50_ value was calculated according to the proportion of wells showing infection at different dilutions.

### 2.6. Animal Grouping and RSV Infection

The experimental unit was defined as a single animal. For both BALB/c mice and cotton rats, 6 animals per group (RSV-infected/PBS control). The sample size (*n* = 6 per group) was determined based on preliminary experiments and validated by post hoc power analysis, using pulmonary viral load as the primary outcome variable. Given the large effect sizes observed in this model, this group size provides sufficient statistical power (1 − β > 0.80) at α = 0.05, while adhering to the 3R principles (Reduction) of animal welfare. A total of 36 female BALB/c mice and 36 female cotton rats (all aged 4–6 weeks) were used in this study. Cotton rats and BALB/c mice in the infected group were infected with RSV via intranasal instillation at a dose of 2.5 × 10^5^ PFU in 50 μL, while control animals received an equal volume of PBS via the same route. The dose was selected according to the titer of our viral stock and validated for its efficacy in preliminary experiments. Specifically, this inoculum consistently induced significant viral replication and lung inflammatory pathology in the model animals while ensuring survival, thereby meeting the observational requirements of this study. After inoculation, the cotton rats and BALB/c mice were placed in lateral recumbency in their cages with free access to food and water. Body weight and temperature were monitored daily until day 5 post-infection. Humane endpoints were predefined; animals were euthanized if showing >20% weight loss, severe lethargy, or respiratory distress. No animals reached these endpoints during the study. On day 3, 5 and 7 post infection, the cotton rats and BALB/c mice were euthanized, and tissues were harvested for subsequent experiments. Potential confounders such as intranasal treatment order or cage location were not explicitly controlled. However, all animals were housed under identical environmental conditions, handled by the same personnel, and monitored on a consistent schedule to minimize baseline variability. Although group allocation was initially unblinded, we implemented a retrospective blinded re-evaluation to minimize observer bias in the semi-quantitative scoring, A random subset of samples (20%) was re-coded and independently assessed by two investigators who were completely unaware of the experimental groups. The reassessment results were highly consistent with the original evaluation, supporting the robustness of the pathological scoring.

### 2.7. Real-Time Quantitative Polymerase Chain Reaction (qRT–PCR)

Total RNA was extracted from tissues using an RNA extraction kit (Vazyme, Nanjing, China) according to the manufacturer’s instructions. Specifically, brain tissues were rapidly harvested without transcardial perfusion and snap-frozen in liquid nitrogen to ensure maximal RNA integrity. RNA purity (A260/A280: 1.9–2.1) was monitored via spectrophotometry, and extraction consistency was ensured by standardized weight-to-buffer ratios and batch processing. qRT–PCR was performed on a CFX96 Touch™ Real–Time PCR Detection System (Bio-Rad Laboratories, Hercules, CA, USA) using the HiScript II One Step qRT–PCR Probe Kit (Vazyme, Nanjing, China). The specific sequences of the RSV primers and probes are shown in Table S1. Representative standard curves were consistently established across different plates (R^2^ > 0.99, efficiency: 95–105%) for the absolute quantitative determination of viral load (Table S2). Samples yielding Ct values outside the reproducible range of the standard curve were considered below the limit of detection and were not assigned quantitative viral RNA copy numbers. Viral RNA copy numbers were determined from Ct values using the standard curve and normalized to the initial tissue weight (copies/mg tissue).

Quantitative analysis was also performed using the HiScript II One Step qRT–PCR SYBR Green Kit (Vazyme, Nanjing, China) with specific primers (Table S3) to measure the mRNA expression of intercellular adhesion molecule-1 (ICAM-1), granulocyte-macrophage colony-stimulating factor (GM-CSF), vascular cell adhesion molecule-1 (VCAM-1), macrophage inflammatory protein-1β (MIP-1β), monocyte chemoattractant protein-1 (CCL2), regulated on activation normal T cell expressed and secreted (CCL5), tumor necrosis factor-α (TNF-α), monocyte chemoattractant protein-3 (CCL7), interferon-induced T cell chemoattractant (CXCL10), interleukin-6 (IL-6), interleukin-1β (IL-1β), and β-actin.

### 2.8. H&E Staining

The animals were deeply anesthetized with an intraperitoneal injection of 1.25% Avertin at a dose of 0.2 mL/10 g body weight, followed by perfusion with 4% paraformaldehyde (Servicebio, Wuhan, China). The lung and brain tissues were then harvested and fixed in 4% paraformaldehyde for 48 h. After dehydration and paraffin embedding, the tissues were sectioned at a thickness of 4 μm. The sections were stained with hematoxylin (Solarbio, Beijing, China) for 3–5 min, washed with running tap water, differentiated in a differentiating solution, washed again with running tap water, incubated in a bluing solution, and finally rinsed with running water. Subsequently, the sections were dehydrated in 95% ethanol for 1 min, stained with eosin (Solarbio, Beijing, China) for 15 s, dehydrated, and then mounted for microscopic examination and image acquisition analysis.

### 2.9. TUNEL Staining

The animals were deeply anesthetized with the same dose and concentration of Avertin as described above, followed by perfusion with 4% paraformaldehyde. The lung tissues were then harvested and fixed in 4% paraformaldehyde for 48 h. After dehydration and paraffin embedding, the tissues were sectioned at a thickness of 4 μm. The sections were deparaffinized, rehydrated, and then subjected to antigen retrieval or digestion with Proteinase K (20 μg/mL) at 37 °C for 30 min. Endogenous peroxidase activity was quenched by incubation with 3% hydrogen peroxide for 10 min at room temperature. The sections were then incubated with the TUNEL reaction mixture (containing TdT enzyme and labeled dUTP) at 37 °C for 60 min in a humidified chamber. After washing, the sections were incubated with Streptavidin-HRP conjugate for 30 min at 37 °C. Positive signals were visualized using a DAB substrate kit (Solarbio, Beijing, China) (development time: 30 s to 5 min), and the nuclei were counterstained with hematoxylin. Finally, the sections were dehydrated, cleared in xylene, and mounted with neutral balsam for microscopic observation and image analysis.

### 2.10. Nissl Staining

The animals were deeply anesthetized with the same dose and concentration of Avertin as described above, followed by perfusion with 4% paraformaldehyde. The brain tissues were then harvested and fixed in 4% paraformaldehyde for 48 h. After dehydration and paraffin embedding, the tissues were sectioned at a thickness of 4 μm. Paraffin sections were deparaffinized and rehydrated through environmentally friendly clearing solutions (Servicebio, Wuhan, China) and graded ethanol, followed by rinsing in running water. Sections were then subjected to Nissl staining for 2–5 min, rinsed with water, and briefly differentiated in 0.1% acetic acid. The differentiation process was monitored under a light microscope and terminated by rinsing with running water. After drying, sections were cleared in xylene, mounted with neutral resin, and examined under a light microscope. Images were acquired for analysis.

### 2.11. Immunohistochemistry

The animals were deeply anesthetized with the same dose and concentration of Avertin as described above, followed by perfusion with 4% paraformaldehyde. The brain tissues were then harvested and fixed in 4% paraformaldehyde for 48 h. After dehydration and paraffin embedding, the tissues were sectioned at a thickness of 4 μm. Tissue sections were deparaffinized in environmentally friendly clearing solutions I, II, and III (10 min each) and dehydrated in absolute ethanol I, II, and III (5 min each). Antigen retrieval was performed in EDTA buffer (Solarbio, Beijing, China) (pH 9.0) by microwaving (5 min high, 5 min stand, 10 min medium-low). After cooling, sections were washed in PBS. Endogenous peroxidase was blocked with 3% H_2_O_2_ (25 min, dark), followed by PBS washes. Sections were blocked with 3% BSA (Solarbio, Beijing, China) (30 min) and incubated with primary antibody [anti-RSV (Abcam, London, UK, catalog # ab43812)] overnight at 4 °C, with negative (no primary) and positive controls included. After washing, HRP-conjugated secondary antibody (Boster, Pleasanton, CA, USA, catalog # BA1055) was applied (50 min, room temperature). DAB was used for color development, monitored microscopically. Sections were counterstained with hematoxylin, dehydrated, cleared in xylene, and mounted for bright-field microscopy.

### 2.12. Immunofluorescence

The animals were deeply anesthetized with the same dose and concentration of Avertin as described above, followed by perfusion with 4% paraformaldehyde. The brain tissues were then harvested and fixed in 4% paraformaldehyde for 48 h. After dehydration and paraffin embedding, the tissues were sectioned at a thickness of 4 μm. The paraffin sections were dewaxed in water, immersed in PBS for 5 min, and boiled in EDTA antigen retrieval solution in a microwave for 25 min. After antigen retrieval, the sections were brought to 20–25 °C, placed in PBS, and agitated on a shaker for three 5 min washes. The sections were then blocked with 3% BSA for 30 min. The primary antibodies [anti-Iba1 (Boster, Pleasanton, CA, USA, catalog # M01394) and anti-GFAP (Abcam, London, UK, catalog # ab68428)] were applied, and the sections were incubated overnight at 4 °C in a humidified chamber. Appropriate negative controls (omission of the primary antibody) and positive controls (tissues known to express the target antigen) were included and processed in parallel for each staining experiment to confirm antibody specificity. After incubation, the sections were brought to room temperature, washed three times with PBS for 5 min each, and then incubated with the corresponding fluorescent secondary antibodies in the dark at room temperature for 50 min. The sections were then washed three times with PBS on a shaker for 5 min each, stained with DAPI (Solarbio, Beijing, China) in the dark at room temperature for 10 min, treated with the quenching solution B (Servicebio, Wuhan, China) for 5 min, and rinsed with running water for 10 min. Finally, the sections were mounted with an anti-fade mounting medium (Solarbio, Beijing, China) and imaged.

### 2.13. Western Blotting

Tissue samples were placed in 1.5 mL Eppendorf tubes and kept on ice. Magnetic beads (Servicebio, Wuhan, China) and RIPA lysis buffer (containing protease inhibitors) (Affinity Biosciences, Cincinnati, OH, USA) were added to the tubes. The samples were homogenized at 4 °C until no clumps remained, then centrifuged at 12,000 rpm for 10 min at 4 °C. The supernatant was collected for BCA protein concentration determination (Seven Biotechnology, Suzhou, China). The protein concentration was adjusted to 8 μg/μL, and then 5 × SDS–PAGE protein loading buffer (Affinity Biosciences, Cincinnati, OH, USA) was added. The samples were boiled for 5 min and stored for later use. SDS–PAGE gels (Affinity Biosciences, Cincinnati, OH, USA) were prepared, and samples were loaded onto the gel. To minimize inter-gel variation, three representative samples per group were loaded onto a single gel to ensure all groups were processed under identical conditions. The gel was run at 80 V for 30 min, then the voltage was increased to 120 V and run for an additional 40–50 min. The proteins were then transferred to a membrane using semi-dry transfer apparatus (Bio-Rad Laboratories, Hercules, CA, USA). The membrane was blocked with a rapid blocking solution (Affinity Biosciences, Cincinnati, OH, USA) at room temperature for 20 min, followed by overnight incubation with primary antibodies at 4 °C. The membrane was washed three times with 1× TBST for 10 min each. Secondary antibodies were added, and the samples were incubated on a shaker at room temperature for 1 h, followed by three 10 min washes with 1× TBST. Finally, the chemiluminescent substrate (Seven Biotechnology, Suzhou, China) was applied to the membrane, and the membrane was imaged using a chemiluminescence imaging system (Bio-Rad Laboratories, Hercules, CA, USA).

### 2.14. Statistical Analysis

No a priori exclusion criteria were established for animals or data points. All animals were included until the end of the experiment, and all data points were used in the analysis. Experimental data are presented as mean ± standard deviation ($\bar{x}$ ± s). Statistical analyses were performed using GraphPad Prism version 10.1.2 (GraphPad Software, Boston, MA, USA). Viral loads were analyzed by two-way ANOVA with Bonferroni’s post hoc test. Histopathological scores were evaluated using the Mann–Whitney U test. For quantitative data (qRT−PCR gene expression, immunofluorescence cell counts, and Western blot densitometry), the unpaired Student’s *t*-test was used for normally distributed data, and the Mann–Whitney U test was used otherwise. False discovery rate (FDR) correction was applied to comparisons across multiple time points and variables to control for type I errors. A *p* value < 0.05 was considered statistically significant. Figures were processed and formatted using Adobe Illustrator 2022 (Adobe Inc., San Jose, CA, USA).

## 3. Results

### 3.1. Susceptibility of Cotton Rats and BALB/c Mice to RSV

**Figure 1:**
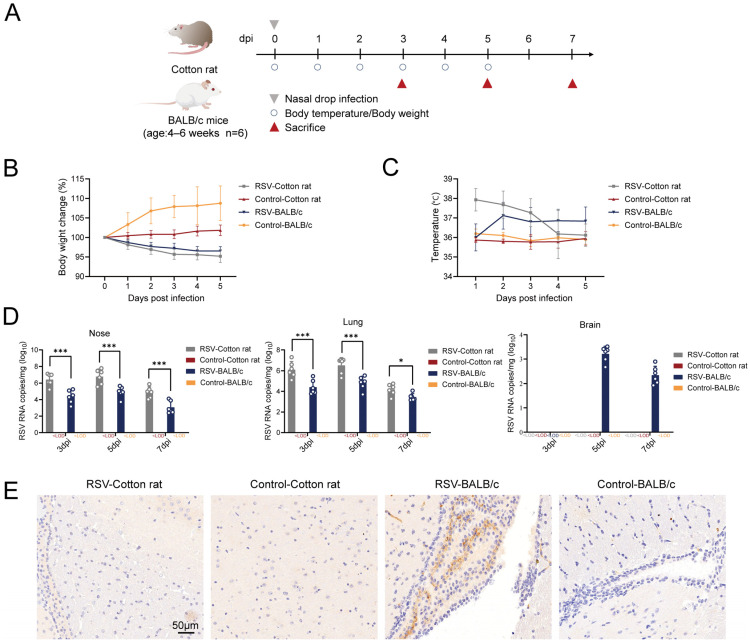
Analysis of the susceptibility of cotton rats and BALB/c mice to RSV. (**A**) Schematic diagram of the experimental design and sampling process for the experiment. (**B**) Weight changes in cotton rats and BALB/c mice after RSV infection (*n* = 6 per group). (**C**) Temperature changes in cotton rats and BALB/c mice after RSV infection (*n* = 6 per group). (**D**) Viral load in the nasal turbinates, lung tissues, and brain tissues of cotton rats and BALB/c mice, as determined by qRT–PCR (*n* = 6 per group). RSV RNA in all control groups and in the brains of RSV-infected cotton rats was below the limit of detection of the qRT–PCR assay. (**E**) Immunohistochemical staining of RSV antigen in brain tissues of cotton rats and BALB/c mice. Data are presented as mean ± SD. * *p* < 0.05, *** *p* < 0.001.

To systematically compare the susceptibility and clinical manifestations of cotton rats and BALB/c mice to RSV, we intranasally infected these two species with RSV on day 0, using PBS of the same volume as a control (Figure 1A). We then monitored their body weight and temperature daily for 5 days after RSV infection. After RSV infection, the body weight of both cotton rats and BALB/c mice decreased continuously, with a greater reduction observed in cotton rats (Figure 1B). The body temperature of both cotton rats and BALB/c mice increased following RSV infection (Figure 1C). Animals were euthanized at 3, 5, and 7 days post-infection (dpi). Tissues, including the nasal turbinates, trachea, lungs, and brain, were collected. RSV RNA was detectable in the nasal turbinates and lung tissues of both cotton rats and BALB/c mice, but not in the trachea. At all examined time points (3, 5, and 7 dpi), the viral loads in the nasal turbinate and lung tissues of RSV-infected cotton rats were significantly higher than those in BALB/c mice infected for the same duration. Notably, the viral loads in both tissues peaked on day 5 post-infection in both species. In BALB/c mice, RSV RNA was detectable in brain tissues at 5 and 7 dpi, though at levels considerably lower than in the respiratory tract. Specifically, the mean viral load was 1.6 × 10^3^ copies/mg at 5 dpi and declined to 2.2 × 10^2^ copies/mg at 7 dpi, while the corresponding pulmonary viral loads were 6.5 × 10^4^ and 2.7 × 10^3^ copies/mg, respectively (Figure 1D). In contrast, RSV RNA was undetectable in the brains of cotton rats at all time points (Figure 1D), indicating levels below the limit of detection. The immunohistochemical staining results for RSV antigens in brain tissue at 5 dpi showed no specific positive staining in the brain sections of RSV-infected cotton rats, consistent with the control group (Figure 1E). In contrast, RSV antigen-positive signals were observed in the brain tissue of RSV-infected BALB/c mice at 5 dpi. Notably, these signals were primarily localized within the epithelial cell layer of the choroid plexus (Figure 1E). This specific distribution suggests that the choroid plexus epithelium may serve as a potential gateway for RSV to enter the brain. Control group BALB/c mice exhibited negative results (Figure 1E).

### 3.2. Pathological Changes in Cotton Rats and BALB/c Mice After RSV Infection

**Figure 2:**
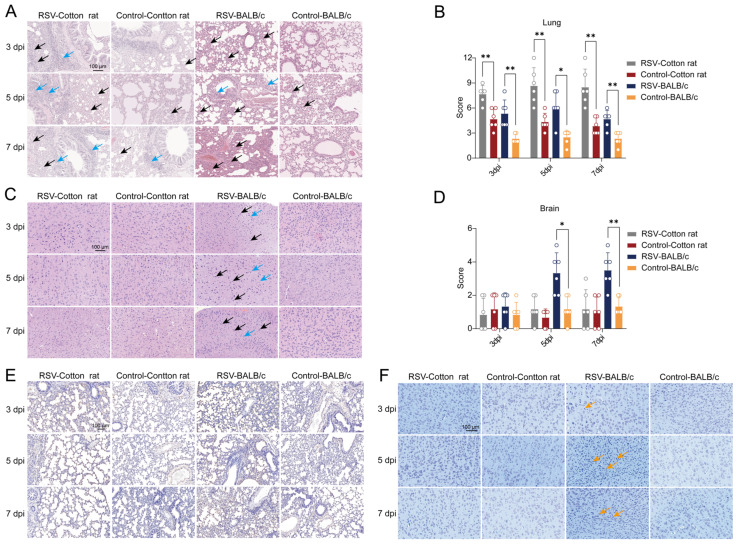
Histopathological examination of lung and brain tissues in cotton rats and BALB/c mice. (**A**) Lung tissue histopathology. Black arrows indicate thickening of alveolar walls and inflammatory cell infiltration; blue arrows indicate lymphocyte infiltration around the bronchi. (**B**) Pathological score for lung tissue. The severity of lesions was evaluated according to the criteria detailed in Table S4. (*n* = 6 per group). (**C**) Brain tissue histopathology. Black arrows indicate shrunken and deeply stained neurons; blue arrows indicate microglial cell infiltrates. (**D**) Pathological score for brain tissue. The severity of lesions was evaluated according to the criteria detailed in Table S4. (*n* = 6 per group). (**E**) Detection of apoptotic cells in lung tissues by TUNEL staining. (**F**) Nissl staining of brain tissues post-RSV infection. Orange arrows indicate neurons with reduced volume and irregular morphology. Data are presented as mean ± SD. * *p* < 0.05, ** *p* < 0.01.

H&E staining was performed on the lung and brain tissues of cotton rats and BALB/c mice. In the control group, both cotton rats and BALB/c mice showed mild interstitial pneumonia in the lung tissues. However, at 3, 5, and 7 dpi, the interstitial pneumonia in the lung tissues of cotton rats exhibited a significant worsening trend, characterized by extensive thickening of the alveolar walls, narrowing or disappearance of alveolar spaces, and massive inflammatory cell infiltration (black arrow); meanwhile, focal infiltration of a small number of lymphocytes was observed around the bronchi, consistent with bronchitis (blue arrow) (Figure 2A). In contrast, although the interstitial pneumonia in the lung tissues of BALB/c mice at the same time points was aggravated compared with their own control group, it only presented as moderate thickening of the alveolar walls over a medium range, narrowing or disappearance of alveolar spaces, and a small number of inflammatory cell infiltration (black arrow); a small number of lymphocytes were also observed around the bronchi, showing mild bronchitis (blue arrow) (Figure 2A). Overall, the extent of lesions and the degree of inflammatory cell infiltration of interstitial pneumonia in the lung tissues of cotton rats were more severe than those in BALB/c mice. Consistent with the above histological observations, the lung pathological scores of cotton rats (evaluated according to the criteria in Table S4) were higher than those of BALB/c mice at all three time points post-RSV infection (Figure 2B). Conversely, in the brain tissues of BALB/c mice post-RSV infection, a moderate proportion of shrunken and deeply stained neurons (25–40% involved area) were observed in the cerebral cortex at 5 and 7 dpi, characterized by indistinct nuclear-cytoplasmic boundaries, enhanced basophilia, and sparse arrangement (black arrow), accompanied by a small number of microglial cell infiltrates (blue arrow) (Figure 2B). However, no such pathological changes were detected in their brain tissues at 3 dpi, which was consistent with the performance of the control group (Figure 2C). In contrast, the brain tissues of cotton rats post-RSV infection showed no significant pathological alterations at all three time points, with only occasional small numbers of shrunken and deeply stained neurons observed in the cortex (black arrow) (Figure 2C). Correspondingly, the brain pathological scores of BALB/c mice at 5 and 7 dpi (evaluated according to the criteria in Table S4) were higher than those of cotton rats at the same time points (Figure 2D).

To assess cellular apoptosis in lung tissues following RSV infection, TUNEL staining was performed. Compared with the control groups, numerous TUNEL-positive nuclei exhibiting characteristic apoptotic morphology were observed in lung tissue sections from both cotton rats and BALB/c mice at 3, 5, and 7 dpi (Figure 2E). The positive signals were predominantly localized to the alveolar walls and peribronchial regions (Figure 2E). Nissl staining was performed on brain tissues to observe morphological changes in neurons. The results showed that in BALB/c mice, neurons in the control group exhibited regular morphology and were arranged densely and orderly; at 5 and 7 dpi, a small number of neurons in the brain tissue showed reduced volume and irregular morphology (Figure 2F). In contrast, no obvious neuronal morphological abnormalities were observed at any time point (3, 5, and 7 dpi) in cotton rats (Figure 2F).

### 3.3. Comparative Analysis of Pulmonary Inflammatory Gene Expression in Cotton Rats and BALB/c Mice

**Figure 3:**
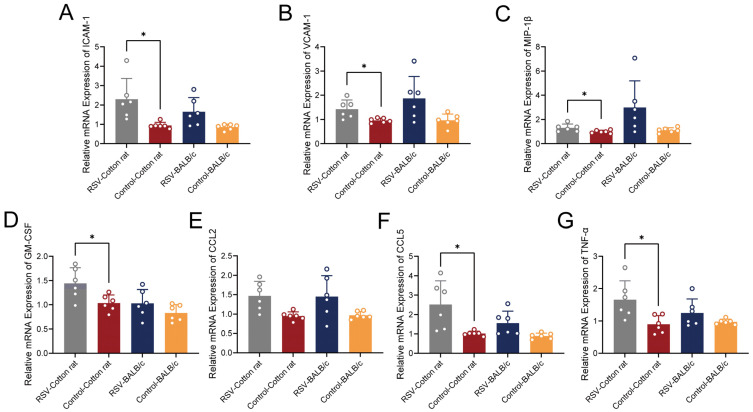
Expression levels of adhesion molecules, chemokines, and cytokines associated with RSV infection in the lung tissues of cotton rats and BALB/c mice. (**A**) ICAM-1, (**B**) VCAM-1, (**C**) MIP-1β, (**D**) GM-CSF, (**E**) CCL2, (**F**) CCL5, and (**G**) TNF-α mRNA levels were quantified by qRT–PCR (*n* = 6 per group). Statistical analyses were performed using appropriate tests, and *p* values were adjusted for multiple comparisons using the FDR method. Data are presented as mean ± SD. * *p* < 0.05.

During the course of RSV infection, various adhesion molecules, chemokines, and cytokines act in concert to modulate immune responses and inflammatory processes. However, there is also a risk of excessive inflammatory reactions [30]. We assessed the mRNA expression profiles of chemokines and cytokines associated with RSV infection at 5 dpi. In cotton rats, RSV infection was associated with increased mRNA levels of ICAM-1, VCAM-1, MIP-1β, GM-CSF, CCL5, and TNF-α in the lungs compared with that in the control animals (Figure 3). In BALB/c mice, ICAM-1 and CCL5 showed a tendency toward increased expression following RSV infection; however, none of the pulmonary inflammatory mediators demonstrated significant expression after FDR correction (Figure 3). In summary, RSV infection was associated with a broader and more robust transcriptional induction of inflammatory mediators in the lungs of cotton rats at the mRNA level.

### 3.4. Comparative Analysis of Brain Inflammatory Gene Expression in Cotton Rats and BALB/c Mice

**Figure 4:**
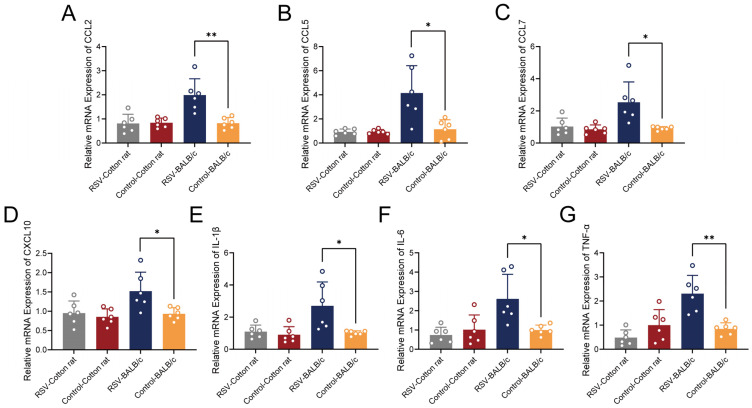
The expression levels of chemokines and cytokines associated with RSV infection in the brain tissues of cotton rats and BALB/c mice. (**A**) CCL2, (**B**) CCL5, (**C**) CCL7, (**D**) CXCL10, (**E**) IL-1β, (**F**) IL-6, and (**G**) TNF-α mRNA levels were quantified by qRT–PCR (*n* = 6 per group). Statistical analyses were performed using appropriate tests, and *p* values were adjusted for multiple comparisons using the FDR method. Data are presented as mean ± SD. * *p* < 0.05, ** *p* < 0.01.

To further assess the impact of RSV infection on the brain, we also detected cytokines and chemokines in the brains of cotton rats and BALB/c mice at 5 dpi. The mRNA expression of CCL2, CCL5, CCL7, CXCL10, TNF-α, IL-6, and IL-1β in the brains of BALB/c mice was significantly elevated compared to that in the control group (Figure 4). All statistical significance shown in Figure 4 was based on FDR-corrected *p* values. These results suggest that RSV infection in BALB/c mice is associated with the transcriptional upregulation of multiple inflammatory mediators in the brain at 5 dpi. In contrast, no significant changes in these transcripts were detected in cotton rats (Figure 4), suggesting a more limited immune response in the brain at this specific time point.

### 3.5. Neuroinflammatory Responses to RSV Infection in BALB/c Mice and Cotton Rats

**Figure 5:**
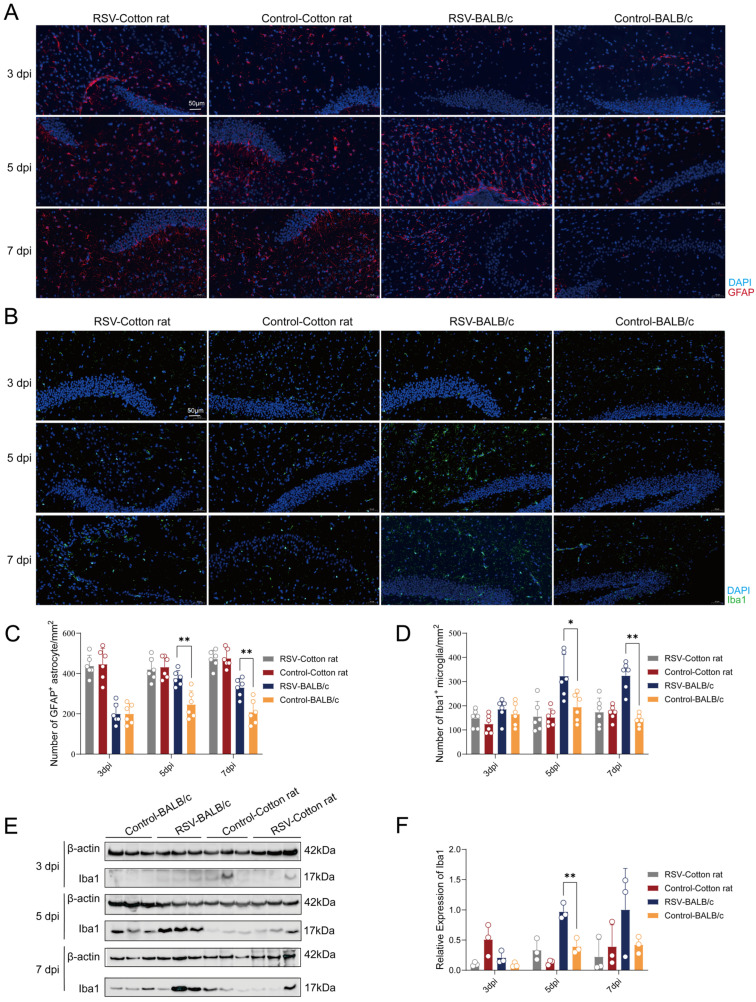
Activation of microglia and astrocytes in the brain tissues of cotton rats and BALB/c mice. (**A**) Immunofluorescence results showing the expression of GFAP in the brain tissues of cotton rats and BALB/c mice at 3, 5, and 7 dpi. (**B**) Immunofluorescence results showing the expression of Iba1 in brain tissues of cotton rats and BALB/c mice at 3, 5, and 7 dpi. (**C**) Quantitative analysis of the number of GFAP+ cells (*n* = 6 per group). (**D**) Quantitative analysis of the number of Iba1+ cells (*n* = 6 per group). (**E**) Western blot results showing the expression of Iba1 protein in the brain tissues of cotton rats and BALB/c mice at 3, 5, and 7 dpi. (**F**) Quantitative analysis of the expression of Iba1 protein (*n* = 3 per group). Statistical analyses were performed using appropriate tests, and *p* values were adjusted for multiple comparisons using the FDR method. Data are presented as mean ± SD. * *p* < 0.05, ** *p* < 0.01.

To further evaluate the impact of RSV infection on the CNS in cotton rats and BALB/c mice, we assessed the expression of glial fibrillary acidic protein (GFAP), an astrocyte activation marker, and ionized calcium-binding adapter molecule 1 (Iba1), a microglial activation marker, using IF and WB analysis. IF indicated that GFAP expression was unchanged in both RSV-infected cotton rats and BALB/c mice at 3 dpi compared with their respective control groups (Figure 5A). However, GFAP expression was upregulated in RSV-infected BALB/c mice compared with that in the control group at 5 and 7 dpi (Figure 5A). Quantitative analysis confirmed a significant increase in GFAP-positive cells in infected BALB/c mice at both time points, whereas no significant difference was observed between infected and control cotton rats at any time point (Figure 5C). Similarly, Iba1 immunofluorescence revealed no microglial activation in either animal model at 3 dpi, but showed evident microglial activation in RSV-infected BALB/c mice at 5 and 7 dpi (Figure 5B). Quantitative data showed a significant increase in Iba1-positive microglia in these mice at 5 and 7 dpi, whereas no significant change was observed in cotton rats at any time point (Figure 5D). Consistent with these findings, WB demonstrated elevated Iba1 protein levels in the brain tissues of infected BALB/c mice at 5 dpi, while no significant change was detected in cotton rats (Figure 5E,F). Taken together, these findings suggest that RSV infection triggers neuroinflammatory responses in BALB/c mice but not in cotton rats.

## 4. Discussion

RSV primarily enters the human body through the upper respiratory tract and is considered to exhibit selective tropism for ciliated airway epithelial cells [31]. However, an increasing body of evidence suggests that RSV infection is not confined to the respiratory tract; the virus can also disseminate to nonrespiratory tissues, thereby inducing a broader range of pathological changes [32]. Neurological complications are among the extrapulmonary manifestations of RSV infection, including encephalitis, seizures, status epilepticus, and central apnea [33]. More importantly, the detection of RSV in the cerebrospinal fluid and brain tissues of patients with neurological abnormalities indicates that RSV can reach the CNS [34,35,36]. A comprehensive and systematic animal model that can present both the typical pulmonary pathological features of RSV infection and accompanying neurological lesions is lacking. The cotton rat model, which is commonly used for evaluating RSV vaccines and drugs, and the BALB/c mouse model, which is frequently employed for RSV mechanism studies, were selected as the subjects of this research to explore the differences in pulmonary and brain tissue pathology following RSV infection in these two models, weigh their advantages and disadvantages, and provide a practical reference for the selection of models in future related studies.

After infection with RSV at a dose of 2.5 × 10^5^ PFU, both cotton rats and BALB/c mice exhibited changes in body weight and temperature, with more pronounced alterations observed in cotton rats, indicating their higher sensitivity to the virus. Tissue samples from the nasal turbinates, trachea, lungs, and brains were collected at 3, 5, and 7 dpi. qRT–PCR analysis revealed that the viral loads in both the nasal turbinates and lungs of cotton rats were significantly higher than those in BALB/c mice at all time points examined. Furthermore, the viral loads in the respiratory tract peaked on day 5 post-infection in both species. This finding suggests that the respiratory tract of cotton rats is more susceptible to RSV, a phenomenon previously confirmed in studies [10,11]. Subsequently, we assessed the expression levels of adhesion molecules, chemokines, and other cytokines in the lung tissues. In the lungs of cotton rats, the mRNA expression levels of VCAM-1, ICAM-1, GM-CSF, MIP-1β, CCL5, and TNF-α were all significantly elevated at 5 dpi. In contrast, RSV infection in BALB/c mice was associated with a more limited induction of pulmonary inflammatory mediators, with trends toward increased expression of ICAM-1 and CCL5 that did not reach significance after correction for multiple comparisons. These findings should therefore be interpreted with caution, as they may reflect biological variability or limited statistical power rather than definitive inflammatory activation. RSV directly targets airway epithelial cells, triggering the release of pro-inflammatory cytokines such as TNF-α and GM-CSF [37,38,39,40,41]. Concurrently, RSV infection activates alveolar macrophages, which constitute a significant source of TNF-α during the inflammatory response [41,42,43,44]. Subsequently, TNF-α activates pulmonary vascular endothelial cells, inducing their high expression of ICAM-1 and VCAM-1, enabling immune cell adhesion and extravasation [45]. Meanwhile, TNF-α and GM-CSF work in concert on epithelial cells and macrophages, stimulating the production of high levels of chemokines, including CCL2, which primarily recruits monocytes, and CCL5 and MIP-1β, which mainly attract lymphocytes and NK cells [46,47]. Ultimately, monocytes, NK cells, and lymphocytes attracted by chemokines, along with neutrophils recruited by GM-CSF, use ICAM-1 and VCAM-1 expressed on endothelial cells as anchor points to cross the vascular wall and infiltrate the lung tissue in large numbers [48,49,50,51]. These highly activated immune cells, in their attempt to clear the virus, release toxic substances such as reactive oxygen species and proteases, leading to lung tissue damage and, ultimately, pneumonia [52]. In summary, while these mediators appear to synergistically promote pulmonary inflammation at the transcriptional level, further protein-level validation is warranted to confirm their functional impact. The H&E staining results of this study demonstrate that, following RSV infection (3, 5, and 7 dpi), the lung tissues of cotton rats exhibit more severe interstitial pneumonia and bronchitis compared with those of BALB/c mice. Concurrent TUNEL staining further revealed that TUNEL-positive apoptotic nuclei were predominantly localized in the alveolar walls and peribronchial regions of both species, with distinct positive signals consistently detected across all three time points. In conclusion, these more pronounced pathological features likely underlie why cotton rats have become the ideal model for studying RSV pulmonary infection.

Interestingly, despite the high viral copy numbers generated in the lungs of cotton rats, RSV RNA was not detected in their brain tissues by qRT–PCR, with all samples remaining below the limit of detection. In contrast, although the viral copy numbers in the lungs of BALB/c mice were significantly lower than those in cotton rats, RSV RNA was detectable in their brain tissues at both 5 and 7 dpi, but not at 3 dpi. Immunohistochemical analysis further indicated that RSV antigen signals were primarily localized to the epithelial cells of the choroid plexus in the ventricular region of BALB/c mice. This specific localization suggests that the choroid plexus epithelium may act as a potential interface for RSV to access the CNS, although the precise mechanisms of viral entry and its potential interaction with parenchymal cells remain to be fully elucidated. H&E staining of BALB/c mice revealed neuronal shrinkage, nuclear pyknosis, and sparse arrangement in the cerebral cortex at 5 and 7 dpi, accompanied by mild microglial activation. These findings were corroborated by Nissl staining, which further identified neurons with reduced volume and irregular contours at these time points. Conversely, brain tissues from cotton rats exhibited no appreciable pathological alterations at any time point, with only rare, isolated shrunken neurons observed. The CNS response to infection and injury typically involves widespread activation of glial cells, a hallmark of neuroinflammation [53]. In this study, RSV infection in BALB/c mice resulted in a distinct neuroinflammatory response at 5 and 7 dpi, characterized by a marked upregulation of the astrocyte marker GFAP and the microglial marker Iba1. No significant differences in these markers were observed at 3 dpi. In contrast, no significant glial cell activation was observed in cotton rats following infection, suggesting a more limited CNS response to RSV infection in this model.

We evaluated various cytokines to further explore the impact of RSV infection on the brains of BALB/c mice. The mRNA expression levels of CCL2, CCL5, CCL7, CXCL10, TNF-α, IL-6, and IL-1β were significantly increased in the brain tissues at 5 dpi, suggesting the induction of a neuroinflammatory response at the transcriptional level. Previous studies have shown that CCL2 and CCL5 are classical inflammatory chemokines [54,55,56]. We hypothesize that RSV infection could potentially alter the permeability of the blood–brain barrier in BALB/c mice to large molecules (CCL2, CCL5, CCL7, and CXCL10), a process that might facilitate the recruitment of immune cells into the CNS. In addition, RSV infection may be associated with an increase in the expression of pro-inflammatory cytokines (IL-1β, IL-6, and TNF-α) in the brain, thereby contributing to potential CNS inflammation [20,57,58]. The neuroinflammatory signals observed in BALB/c mice may also be influenced by the lung–brain axis [26]. Previous studies have established that RSV-induced pulmonary inflammation can systemically disseminate pro-inflammatory signals, with cytokines like TNF-α and IL-6 playing pivotal roles in modulating neuroinflammation and BBB integrity [26,59]. In our study, the pronounced elevation of pulmonary TNF-α in cotton rats did not translate into CNS inflammation, highlighting a critical, species-specific disconnection in this axis, which could be speculated to be influenced by differences in the BBB permeability. An in vitro comparative study suggested that intrinsic characteristics of the BBB, such as the expression of tight junction proteins and baseline barrier integrity, can differ notably between common rodent models such as mice and rats [60], and even across different laboratory mouse strains [61]. The species-specific divergence in CNS responses might be linked to differences in type I interferon (IFN) responses, a key antiviral defense mechanism that RSV can suppress through viral proteins such as NS1, NS2, and the G glycoprotein [62,63]. Although IFN responses were not directly measured in the present study, it remains hypothetical that cotton rats mount a more robust systemic IFN response than BALB/c mice, which may effectively restrict the systemic dissemination of the virus [64]. The close relationships and specific molecular mechanisms underlying RSV infection and neuroinflammation are important research directions and the foci of our future studies.

The present study has several limitations that should be acknowledged. The analyses were limited to acute time points following infection, meaning that potential long-term neurological or behavioral consequences were not assessed. In addition, the absence of prospective blinding represents a limitation that may introduce observer bias, particularly in semi-quantitative analyses. To mitigate this concern, we conducted a retrospective blinded re-scoring of a randomly selected subset of samples. The high concordance between the original and blinded assessments suggests that the impact of this bias on the main conclusions is likely minimal. Nevertheless, future studies should incorporate strict prospective blinding to further strengthen methodological rigor. While the sample size was adequate for detecting significant differences between groups, it may have been insufficient to identify more subtle effects, thereby limiting the generalizability of our findings to some extent. These limitations warrant further investigation in future studies.

## 5. Conclusions

In summary, our findings indicate that the cotton rat may be more advantageous than the BALB/c mouse for investigating RSV pulmonary infection and evaluating related vaccines and drugs. However, under the conditions of this study, neither RSV RNA nor antigens were detected in brains of cotton rats, nor were associated neuroinflammatory responses observed, indicating the limitations of this model for studying RSV-related CNS complications. In contrast, RSV-infected BALB/c mice not only exhibited respiratory tract infection but also detectable viral RNA and antigens in their brain tissue, accompanied by clear neuroinflammatory responses. Therefore, the BALB/c mouse model may provide a useful experimental system for investigating RSV-associated CNS inflammatory responses under experimental infection conditions, although the clinical relevance of these findings requires further investigation. The research data on RSV infection in these two animal models provide an important reference for researchers in relevant fields.

## Data Availability

The data supporting the conclusions of this article are included within the article or are available from the authors upon reasonable request.

## Supplementary Materials

The following supporting information can be downloaded at: https://www.mdpi.com/article/10.3390/v18030382/s1, Table S1: qPCR primer sequences; Table S2: Standard Curve; Table S3: qPCR primer sequences; Table S4: Histopathological Score.

## Abbreviations

ALRTI	Acute lower respiratory tract infections
BBB	Blood–Brain Barrier
CCL2	C-C Motif Chemokine Ligand 2
CCL5	C-C Motif Chemokine Ligand 5
CCL7	C-C Motif Chemokine Ligand 7
CNS	Central Nervous System
CPE	Cytopathic Effect
Ct	Cycle Threshold
CTL	Cytotoxic T Lymphocyte
CXCL10	C-X-C Motif Chemokine Ligand 10
DAPI	4′,6-Diamidino-2-Phenylindole
DMEM–F12	Dulbecco’s Modified Eagle Medium–Nutrient Mixture F-12
GM-CSF	Granulocyte–Macrophage Colony-Stimulating Factor
VCAM-1	Vascular Cell Adhesion Molecule-1
hRSV	Human Respiratory Syncytial Virus
HRP	Horseradish Peroxidase
Iba1	Ionized Calcium-Binding Adapter Molecule 1
ICAM-1	Intercellular Adhesion Molecule-1
IF	Immunofluorescence
IgG	Immunoglobulin G
IL-1β	Interleukin-1β
IL-6	Interleukin-6
MIP-1β	Macrophage Inflammatory Protein-1β
MOI	Multiplicity Of Infection
PFU	Plaque-Forming Units
RIPA	Radioimmunoprecipitation Assay
RSV	Respiratory Syncytial Virus
TBST	Tris-Buffered Saline with Tween 20
TCID_50_	Median Tissue Culture Infectious Dose
TNF-α	Tumor Necrosis Factor-α
