# Supplementary material for: Pathological Characteristics of the Lung and Brain in Cotton Rats and BALB/c Mice Infected with Respiratory Syncytial Virus
Source: Viruses. 2026 Mar 18;18(3):382. doi: 10.3390/v18030382 (PMC13030870; doi:10.3390/v18030382)
Supplement: Supplementary file 1 [file viruses-18-00382-s001.zip › viruses-4169880-supplementary/Table S1 qPCR primer sequences.pdf]

Table S1. qPCR primer sequences

Gene name	Forward primer	Reverse primer
RSV	GTACTAATTTAGCWGGACATTGGATTC	ACWTTCAAATTRATGAACATATGATCAGT
RSV probe	CAACTTATGAAAGATTC	
