# Supplementary material for: Pathological Characteristics of the Lung and Brain in Cotton Rats and BALB/c Mice Infected with Respiratory Syncytial Virus
Source: Viruses. 2026 Mar 18;18(3):382. doi: 10.3390/v18030382 (PMC13030870; doi:10.3390/v18030382)
Supplement: Supplementary file 1 [file viruses-18-00382-s001.zip › viruses-4169880-supplementary/Table S3 qPCR primer sequences.pdf]

Table S3. qPCR primer sequences

Gene name	Forward primer	Reverse primer
ICAM-1	AGTCGTCCGCTTCCGCTACC	AGGGTGAGGTCCTTGCCTACTTG
VCAM-1	TGTGCTGCTATTGGCTGTGACTC	CCCTCATTCCTTACCCCATTG
MIP-1β	TGCTCGTGGCTGCCTTCTG	GGTGTAAGAGAAACAGCAGGAAGTG
GM-CSF	CTTGAACATGACAGCCAGCTA	AATCCGCATAGGTGGTAACTTGTG
CCL2	GCATCTGCCCTAAGGTCTTCAGC	GTTCACTGTCACACTGGTCACTCC
CCL5	GACACCACTCCCTGCTGCTTTG	CTCTGGGTTGGCACACACTTGG
CCL7	TGCTCATAGCCGCTGCTTTCAG	CTTCCCAGGGACACCGACTACTG
CXCL10	TCCGCTGCAACTGCATCCATATC	GGATAGGCTCGCAGGGATGATTTC
IL-1β	TCGCAGCAGCACATCAACAAG	TCCACGGGAAAGACACAGGTAG
IL-6	GTTGCCTTCTTGGGACTGATGC	GGTATCCTCTGTGAAGTCTCCTCTC
TNF-α	CTGTAGCCCACGTCGTAGC	TTGAGATCCATGCCGTTG
β-actin	GCTCTCCCTCACGCCATCC	GTCACGCACGATTTCCCTCTC
