# Supplementary material for: Pathological Characteristics of the Lung and Brain in Cotton Rats and BALB/c Mice Infected with Respiratory Syncytial Virus
Source: Viruses. 2026 Mar 18;18(3):382. doi: 10.3390/v18030382 (PMC13030870; doi:10.3390/v18030382)
Supplement: Supplementary file 1 [file viruses-18-00382-s001.zip › viruses-4169880-supplementary/Table S4 Histopathological Score.pdf]

Evaluation Criteria

Lung

Grade (Numerical)	Category	Morphological Criteria Description
0	Within Normal Range	The lung tissue surface is smooth; the alveolar walls are thin, composed of a single layer of epithelium with a clear structure; the alveolar lumens are patent; the interstitial and bronchial structures are unremarkable; no perivascular inflammatory cell aggregation is observed; no inflammatory changes are noted; the involved area is less than 5%.
1	Very Mild	Focal mild thickening of alveolar walls with mild stenosis or focal obliteration of alveolar lumens; accompanied by a small number of inflammatory cell infiltration; mild scattered perivascular inflammatory cells present; bronchial structures are unremarkable; no obvious interstitial abnormalities are noted; the involved area ranges from 5% to 10%.
2	Mild	Moderate thickening of alveolar walls with partial stenosis or obliteration of alveolar lumens; prominent inflammatory cell infiltration is evident; moderate perivascular inflammatory cell aggregation present; the involved area ranges from 10% to 25%.
3	Moderate	Focal moderate thickening of alveolar walls with stenosis or focal obliteration of alveolar lumens; accompanied by abundant inflammatory cell infiltration; prominent perivascular cuffing infiltration present; the involved area ranges from 25% to 40%.
4	Severe	Extensive thickening of alveolar walls with widespread stenosis or obliteration of alveolar lumens; accompanied by massive inflammatory cell infiltration; massive perivascular cuffing infiltration present; mild necrosis of bronchial epithelial cells is observed, with nuclear pyknosis and cytoplasmic vacuolization; the involved area ranges from 40% to 60%.

5	Extremely Severe	Extensive structural destruction with indiscernible alveolar architecture; massive inflammatory infiltration accompanied by necrosis or hemorrhage; diffuse perivascular cuffing infiltration present; severe lung tissue damage; the involved area is greater than 60%.

Brain

Grade (Numerical)	Category	Morphological Criteria Description
0	Within Normal Range	Neurons with regular morphology, arranged in an orderly and dense pattern; large and round nuclei with scanty chromatin and prominent nucleoli; no microglial infiltration, necrosis or hemorrhage observed; the involved area is less than 5%.
1	Very Mild	Focal mild and scattered microglial infiltration; neurons generally unremarkable with well-preserved orderly arrangement; no obvious pyknosis or necrosis observed; the involved area ranges from 5% to 10%.
2	Mild	A small number of neurons showing pyknosis and hyperchromasia, with indistinct nuclear-cytoplasmic boundaries and slightly sparse arrangement; accompanied by a small amount of microglial infiltration; no necrosis or hemorrhage observed; the involved area ranges from 10% to 25%.
3	Moderate	A moderate number of neurons exhibiting pyknosis with hyperchromatic nuclei and markedly sparse arrangement; mild aggregation of microglia; focal necrosis or mild hemorrhage may be present; the involved area ranges from 25% to 40%.
4	Severe	Massive neurons showing pyknosis, hyperchromasia and disorganized arrangement; prominent microglial infiltration; obvious necrosis accompanied by focal hemorrhage; the involved area ranges from 40% to 60%.
5	Extremely Severe	Diffuse severe neuronal pyknosis or loss; extensive microglial infiltration; massive necrosis accompanied by widespread hemorrhage; severe destruction of brain tissue structure; the involved area is greater than 60%.

Cotton Rats and BALB/c Mice Lung Histopathological Score

3dpi

Lesions / No.	Alveolar Wall Thickening	Inflammatory Cell Infiltration	Alveolar Dilation	Perivascular Cuffing	Total Score
RSV-Cotton rat 1	4	4	0	1	9
RSV-Cotton rat 2	3	3	0	2	8
RSV-Cotton rat 3	4	3	0	1	8
RSV-Cotton rat 4	4	4	0	0	8
RSV-Cotton rat 5	3	4	0	0	7
RSV-Cotton rat 6	4	2	0	1	6
Control-Cotton rat 1	3	3	0	0	6
Control-Cotton rat 2	2	2	1	1	6
Control-Cotton rat 3	3	2	0	0	5
Control-Cotton rat 4	2	2	0	0	4
Control-Cotton rat 5	2	1	0	1	4
Control-Cotton rat 6	2	1	0	0	3
RSV-BALB/c 1	4	4	0	0	8
RSV-BALB/c 2	4	2	0	0	6
RSV-BALB/c 3	3	2	0	1	6
RSV-BALB/c 4	2	2	0	0	4
RSV-BALB/c 5	2	2	0	0	4
RSV-BALB/c 6	3	1	0	0	4
Control-BALB/c 1	2	1	0	0	3

Control-BALB/c 2	2	1	0	0	3
Control-BALB/c 3	2	0	0	0	2
Control-BALB/c 4	2	0	0	0	2
Control-BALB/c 5	2	0	0	0	2
Control-BALB/c 6	1	1	0	0	2

5dpi

Lesions / No.	Alveolar Wall Thickening	Inflammatory Cell Infiltration	Alveolar Dilation	Perivascular Cuffing	Total Score
RSV-Cotton rat 1	5	5	0	2	12
RSV-Cotton rat 2	4	4	2	0	10
RSV-Cotton rat 3	4	3	0	2	9
RSV-Cotton rat 4	3	3	0	2	8
RSV-Cotton rat 5	3	3	0	1	7
RSV-Cotton rat 6	4	2	0	0	6
Control-Cotton rat 1	3	2	0	1	6
Control-Cotton rat 2	2	2	1	0	5
Control-Cotton rat 3	2	2	0	0	4
Control-Cotton rat 4	2	1	0	0	4
Control-Cotton rat 5	2	1	0	1	4
Control-Cotton rat 6	2	1	0	0	3
RSV-BALB/c 1	4	4	0	0	8
RSV-BALB/c 2	4	4	0	0	8
RSV-	4	2	0	0	6

BALB/c 3					
RSV-BALB/c 4	4	2	0	0	6
RSV-BALB/c 5	3	3	0	0	6
RSV-BALB/c 6	2	1	0	0	3
Control-BALB/c 1	2	1	0	0	3
Control-BALB/c 2	2	1	0	0	3
Control-BALB/c 3	3	0	0	0	3
Control-BALB/c 4	3	0	0	0	3
Control-BALB/c 5	2	0	0	0	2
Control-BALB/c 6	2	0	0	0	2

7dpi

Lesions / No.	Alveolar Wall Thickening	Inflammatory Cell Infiltration	Alveolar Dilation	Perivascular Cuffing	Total Score
RSV-Cotton rat 1	5	5	0	2	12
RSV-Cotton rat 2	4	4	0	2	10
RSV-Cotton rat 3	3	3	2	0	8
RSV-Cotton rat 4	3	3	0	2	8
RSV-Cotton rat 5	3	2	0	2	7
RSV-Cotton rat 6	4	2	0	0	6
Control-Cotton rat 1	2	2	0	1	5
Control-Cotton rat 2	2	1	1	0	4
Control-Cotton rat 3	1	2	0	0	3
Control-Cotton rat 4	2	1	0	0	3

Control-Cotton rat 5	2	0	0	1	3
Control-Cotton rat 6	2	0	0	0	2
RSV-BALB/c 1	3	3	0	0	6
RSV-BALB/c 2	3	2	0	0	5
RSV-BALB/c 3	3	2	0	0	5
RSV-BALB/c 4	2	2	0	1	5
RSV-BALB/c 5	2	2	0	0	4
RSV-BALB/c 6	2	1	0	0	3
Control-BALB/c 1	3	0	0	0	3
Control-BALB/c 2	2	1	0	0	3
Control-BALB/c 3	2	1	0	0	3
Control-BALB/c 4	2	0	0	0	2
Control-BALB/c 5	2	0	0	0	2
Control-BALB/c 6	1	0	0	0	1

Cotton Rats and BALB/c Mice Brain Histopathological Score

3dpi

Lesions / No.	Neuronal Pyknosis	Gilal Cell Infiltration	Necrosis	Hemorrhage	Total Score
RSV-Cotton rat 1	2	0	0	0	2
RSV-Cotton rat 2	2	0	0	0	2
RSV-Cotton rat 3	1	0	0	0	1
RSV-Cotton rat 4	0	0	0	0	0
RSV-Cotton rat 5	0	0	0	0	0

RSV-Cotton rat 6	0	0	0	0	0
Control-Cotton rat 1	2	0	0	0	2
Control-Cotton rat 2	2	0	0	0	2
Control-Cotton rat 3	1	1	0	0	2
Control-Cotton rat 4	1	0	0	0	1
Control-Cotton rat 5	0	0	0	0	0
Control-Cotton rat 6	0	0	0	0	0
RSV-BALB/c 1	2	0	0	0	2
RSV-BALB/c 2	1	1	0	0	2
RSV-BALB/c 3	1	1	0	0	2
RSV-BALB/c 4	1	0	0	0	1
RSV-BALB/c 5	1	0	0	0	1
RSV-BALB/c 6	0	0	0	0	0
Control-BALB/c 1	2	0	0	0	2
Control-BALB/c 2	1	0	0	0	1
Control-BALB/c 3	1	0	0	0	1
Control-BALB/c 4	1	0	0	0	1
Control-BALB/c 5	0	0	0	0	0
Control-BALB/c 6	0	0	0	0	0

5 dpi

Lesions / No.	Neuronal Pyknosis	Gilal Cell Infiltration	Necrosis	Hemorrhage	Total Score
RSV-Cotton rat 1	2	0	0	0	2

RSV-Cotton rat 2	2	0	0	0	2
RSV-Cotton rat 3	1	0	0	0	1
RSV-Cotton rat 4	1	0	0	0	1
RSV-Cotton rat 5	1	0	0	0	1
RSV-Cotton rat 6	0	0	0	0	0
Control-Cotton rat 1	1	0	0	0	1
Control-Cotton rat 2	0	1	0	0	1
Control-Cotton rat 3	1	0	0	0	1
Control-Cotton rat 4	1	0	0	0	1
Control-Cotton rat 5	0	0	0	0	0
Control-Cotton rat 6	0	0	0	0	0
RSV-BALB/c 1	3	2	0	0	5
RSV-BALB/c 2	2	2	0	0	4
RSV-BALB/c 3	3	1	0	0	4
RSV-BALB/c 4	2	1	0	0	3
RSV-BALB/c 5	2	0	0	0	2
RSV-BALB/c 6	2	0	0	0	2
Control-BALB/c 1	2	0	0	0	2
Control-BALB/c 2	1	1	0	0	2
Control-BALB/c 3	1	0	0	0	1
Control-BALB/c 4	1	0	0	0	1
Control-BALB/c 5	1	0	0	0	1

Control-BALB/c 6	0	0	0	0	0

7 dpi

Lesions / No.	Neuronal Pyknosis	Gilal Cell Infiltration	Necrosis	Hemorrhage	Total Score
RSV-Cotton rat 1	2	1	0	0	3
RSV-Cotton rat 2	2	0	0	0	2
RSV-Cotton rat 3	1	0	0	0	1
RSV-Cotton rat 4	1	0	0	0	1
RSV-Cotton rat 5	0	0	0	0	0
RSV-Cotton rat 6	0	0	0	0	0
Control-Cotton rat 1	2	0	0	0	2
Control-Cotton rat 2	2	0	0	0	2
Control-Cotton rat 3	1	0	0	0	1
Control-Cotton rat 4	1	0	0	0	1
Control-Cotton rat 5	0	0	0	0	0
Control-Cotton rat 6	0	0	0	0	0
RSV-BALB/c 1	3	2	0	0	5
RSV-BALB/c 2	2	2	0	0	4
RSV-BALB/c 3	3	1	0	0	4
RSV-BALB/c 4	2	1	0	0	3
RSV-BALB/c 5	2	1	0	0	3
RSV-BALB/c 6	2	0	0	0	2
Control-BALB/c 1	1	1	0	0	2

Control-BALB/c 2	2	0	0	0	2
Control-BALB/c 3	1	0	0	0	1
Control-BALB/c 4	1	0	0	0	1
Control-BALB/c 5	1	0	0	0	1
Control-BALB/c 6	1	0	0	0	1
